# Comparative Study of Leaf and Rootstock Aqueous Extracts of *Foeniculum vulgare* on Chemical Profile and *In Vitro* Antioxidant and Antihyperglycemic Activities

**DOI:** 10.1155/2020/8852570

**Published:** 2020-09-01

**Authors:** Karima Sayah, Nasreddine El Omari, Mourad Kharbach, Abdelhakim Bouyahya, Rabie Kamal, Ilias Marmouzi, Yahia Cherrah, My El Abbes Faouzi

**Affiliations:** ^1^Biopharmaceutical and Toxicological Analysis Research Team, Laboratory of Pharmacology and Toxicology, Faculty of Medicine and Pharmacy, Mohammed V University, Rabat, Morocco; ^2^Laboratory of Histology, Embryology, and Cytogenetic, Faculty of Medicine and Pharmacy, Mohammed V University, Rabat, Morocco; ^3^Laboratory of Human Pathology Biology, Faculty of Sciences, Genomic Center of Human Pathology, Faculty of Medicine and Pharmacy, Mohammed V University, Rabat, Morocco; ^4^Pharmacodynamy Research Team ERP, Laboratory of Pharmacology and Toxicology, Faculty of Medicine and Pharmacy, Mohammed V University, Rabat, Morocco

## Abstract

*Foeniculum vulgare* is a medicinal plant used in Moroccan folk medicine to treat several diseases such as diabetes. The aim of this study was to determine the phenolic bioactive compounds and to evaluate the antioxidant and antihyperglycemic activities of *Foeniculum vulgare* leaf and rootstock extracts. Phenolic compounds of *F. vulgare* rootstock and leaf extracts were determined using HPLC-DAD-QTOFMS analysis. The antioxidant activity was evaluated using 1,1-diphenyl-2-picrylhydrazyl (DPPH) and 2,2'-azinobis-(3-ethylbenzothiazoline-6-sulfonic acid) (ABTS^•+^) radicals. Moreover, the *in vitro* antihyperglycemic effects were tested by measuring the inhibition of *α*-amylase and *α*-glucosidase activities. HPLC-DAD-QTOFMS analysis identified thirty-two phenolic components in both leaf and rootstock extracts. Caffeic acid, quinic acid, and chlorogenic acid were the major compounds of *F. vulgare* leaf extract (FVLE), while the main compound of *F. vulgare* rootstock extracts (FVRE) was quinic acid. In the DPPH assay, *F. vulgare* leaf extract showed important antioxidant activity (IC_50_ = 12.16 ± 0.02 *μ*g/mL) than *F. vulgare* rootstock extract (IC_50_ = 34.36 ± 0.09 *μ*g/mL). Moreover, fennel leaf extracts revealed also the most powerful antioxidant activity (IC_50_ = 22.95 ± 0.4 *μ*g/mL) in the ABTS assay. The *in vitro* antihyperglycemic activity showed that *F. vulgare* rootstock extract exhibited a remarkable inhibitory capacity (IC_50_ = 194.30 ± 4.8 *μ*g/mL) of *α*-amylase compared with *F. vulgare* leaf extract (IC_50_ = 1026.50 ± 6.5 *μ*g/mL). Furthermore, the inhibition of *α*-glucosidase was more importantly with *F. vulgare* rootstock (IC_50_ of 165.90 ± 1.2 *μ*g/mL) than *F. vulgare* leaf extracts (203.80 ± 1.3 *μ*g/mL). The funding of this study showed that *F. vulgare* rootstock and leaf extracts presented several phenolic compounds and showed important antioxidant and antidiabetic effects. We suggest that the identified molecules are responsible for the obtained activities. However, further studies focusing on the isolation and the determination of antioxidant and antidiabetic effects of *F. vulgare* rootstock and leaf main compounds are required.

## 1. Introduction

Recourse to the use of medicinal plants in the treatment of diseases has been common since antiquity [1, 2]. The low risk of side effects allows them to be considered a good alternative to synthesized products [3, 4]. Nowadays, the prevalence of diabetes is constantly increasing; it is a chronic disease characterized by a deficiency of endogenous insulin secretion by pancreatic *β*-cells and/or an altered action of this hormone [5]. Recently, the treatment of diabetes has attracted great interest whether in traditional medicine or scientific research. Indeed, several species of medicinal plants are scientifically evaluated and traditionally used for this purpose [6–9]. The antidiabetic properties of these plants are attributed to the presence of certain chemical compounds such as phenolic acids, flavonoids, and terpenoids [10]. Moreover, this activity may depend on several mechanisms aimed at stimulating the secretion of insulin by *β*-cells, the reduction of insulin resistance, the prevention against oxidative stress, and the inhibition of sugar digestive enzymes especially *α*-glucosidase and *α*-amylase [11–13].

Among the medicinal plants, *Foeniculum vulgare* L., Mill (Apiaceae), commonly known as fennel, a very common plant in the Mediterranean region [14, 15]. It is used in traditional medicine to cure a variety of diseases, and its fruits were used as culinary spices [16]. The objective of the present study is to identify the phenolic composition of aqueous extracts prepared from *F. vulgare* leaves and rootstocks and to evaluate their *in vitro* antihyperglycemic and antioxidant properties.

## 2. Materials and Methods

### 2.1. Preparation of Plant Extracts

*Foeniculum vulgare* was collected in July 2017 from Rabat-Zaer region in Morocco. The rootstock and leaves were cleaned and were then used for further investigation. Plant material (100 g) was extracted with water using the decoction method for 1 h. The obtained extracts were then filtered on Whatman paper, and the filtrate obtained was evaporated under reduced pressure, using a rotary evaporator.

### 2.2. Chemical Composition

Chemical composition was determined using HPLC-DAD/TOFMS analysis of phenolic profiling according to the previous work done by Marmouzi et al. [17] with some modifications. In brief, an Agilent 1100 model series of liquid chromatography apparatus (HPLC, Agilent Technologies, Wilmington, DE, USA) was consisting of binary pump (G1312-A) and autosampler (G1330-B) and equipped to diode-array detector (G1315-B). The system was coupled to a time-of-flight (TOF) and a mass spectroscopic detector (MS) equipped with an electrospray ionizer source (ESI) (Micromass Quattro Micro, Agilent Technologies, Wilmington, DE, USA). The operational conditions were as follows: negative mode; range *m/z* 50–1200; nitrogen was used as desolvation and cone gas; cone gas flow rate at 30.0 L/h; desolvation gas flow rate at 350 L/h; cone voltage, 20 V; capillary voltage, 3.0 kV; extractor, 2 V; desolvation temperature 350°C; and source temperature 100°C. The phenolic separation was achieved on a C18 column (2.1 mm × 100 mm × 1.7 *μ*m, Eclipse, XDB/Agilent Zorbax) at a temperature set at 35°C. The mobile phase composed of A (0.1% acetic acid in pure water) and B (0.1% acetic acid in acetonitrile) under a gradient elution (v/v) as follows: 0 min, 10% B; 0–18 min, 10–70% B; 18–20 min, 70–100% B; 20–23 min, 100–100% B; 20–23 min, 100–100% B; 23–25 min, 100–10% B; 25–30 min, 10–10% B at a flow rate of 0.5 mL/min, and 10 *μ*L was injected. The identification of phenolic compounds was performed by comparing their mass spectra fragmentation and retention times with those of pure standards (Sigma-Aldrich, St. Quentin Fallavier, France), while the calibration curves were applied for their quantification. Each polyphenolic extract sample was dissolved in pure water; 10 mg of each extract was dissolved in water (10 mL) and then mixed by vortex and sonicated for 10 min; then, 1 mL was filtered at a syringe filter (PVDF, 0.2 *μ*m) before the HPLC-DAD/TOFMS analysis.

### 2.3. Antioxidant Effect

#### 2.3.1. DPPH Radical Scavenging Activity

Radical scavenging activity of the extracts was measured using the stable-free radical DPPH [6]. In brief, the solution of DPPH (0.2 mM) was prepared in methanol, and 0.5 mL of this solution was added to 2.5 mL of the extract and was allowed to stand at room temperature for 30 min. The absorbance was then read at 517 nm. Trolox was used as reference compound. The radical scavenging activity (RSA) was calculated using the following equation:

(1) $$\begin{array}{c} \text{RSA}\left(\%\right)=\left(\frac{\left(A0-A1\right)}{A0}\right)\times 100, \end{array}$$

where *A*0 is the absorbance of the control reaction and *A*1 is the absorbance of the sample solution. Concentration of the extract required to inhibit 50% of the free radical scavenging activity (IC_50_) was determined.

#### 2.3.2. ABTS Radical Scavenging Activity

The ABTS radical scavenging activity of the plant extracts was estimated using the previously described method [8]. The radical cation ABTS^•+^ was produced through the reaction between 2 mM ABTS and 70 mM potassium persulfate in water. The mixture was stored at room temperature in the dark for 24 h prior use. The ABTS solution was then diluted with methanol to obtain an absorbance of 0.70 at 734 nm. 100 *μ*L of appropriately diluted extracts was added to 2 mL of ABTS solution, and the absorbance was recorded at 734 nm after 1 min incubation at room temperature. Trolox was used as inhibitor standard. ABTS radical scavenging activity was calculated as described in equation (1).

### 2.4. Antihyperglycemic Activity

#### 2.4.1. *α*-Amylase Inhibition Assay

The *α*-amylase inhibition assay was performed using the previously reported method [8]. In brief, 250 *μ*L of various concentrations of the samples and 250 *μ*L of *α*-amylase (240 U/mL, in 0.02 M sodium phosphate buffer, pH 6.9) were mixed and incubated at 37°C for 20 min. A portion (250 *μ*L) of soluble starch (1% (w/v)) (in 0.02 M phosphate buffer, pH 6.9) was added, and the mixture was further incubated at 37°C for 15 min. Finally, 1 mL of dinitrosalicylic acid (DNS) color reagent was added and incubated in a boiling water bath for 10 min to stop the reaction. The mixture was then diluted with 2 mL of distilled water, and the absorbance was measured at 540 nm. Acarbose was used as positive control. The results were expressed as percentage inhibition and calculated using the following formula:

(2) $$\begin{array}{c} \text{inhibition}\left(\%\right)\;=\;\left[\frac{\left(Ac-Acb\right)-\left(As-Asb\right)}{\left(Ac-Acb\right)}\right]\times 100, \end{array}$$

where *Ac* refers to the absorbance of control (enzyme and buffer), *Acb* refers to the absorbance of control blank (buffer without enzyme), *As* refers to the absorbance of sample (enzyme and inhibitor), and *Asb* is the absorbance of sample blank (inhibitor without enzyme).

#### 2.4.2. *α*-Glucosidase Inhibition Assay

The *α*-glucosidase inhibitory activity of the extracts was estimated using *p*-nitrophenyl-*α*-D-glucopyranoside (*p*-NPG) as substrate [18]. 150 *μ*L of extracts at different concentrations were mixed with 100 *μ*L of *α*-glucosidase enzyme solution (0.1 U/ml) prepared in 0.1 M sodium phosphate buffer (pH = 6.7) and incubated at 37°C for 10 min. Then, a portion (200 *μ*L) of 1 mM substrate *p*-NPG was added, and the mixture was incubated at 37°C for 30 min. The reaction was then stopped by the addition of 1 mL of Na_2_CO_3_ (1 M), and the absorbance was recorded at 405 nm. The percentage inhibition of *α*-glucosidase enzyme of different concentrations of extracts was also measured using formula (2) described above, and the IC_50_ values were calculated.

### 2.5. Statistical Analysis

The significance of differences between multiple averages was determined by one-way analysis of variance (ANOVA), followed by Tukey's post hoc test at *p* < 0.05 significance level. Analysis was performed with GraphPad Prism 6.

## 3. Results and Discussion

### 3.1. Chemical Composition

The phenolic chemical compounds of *F. vulgare* extracts were determined by HPLC-DAD-QTOFMS analysis. The results obtained are summarized in Table 1. As listed, thirty-two phenolic components were identified and quantified. The quantification analysis revealed important variabilities between FVLE and FVRE for all measured compounds (Table 1). Caffeic acid, quinic acid, and chlorogenic acid were the major phenolic compounds of FVLE by a percentage rate of 509203 ± 254.60, 14945.61 ± 747.28, and 13810 ± 690.53 *μ*g/mg extract, respectively. Moreover, the main compound of FVRE was quinic acid (29416.42 ± 1470.82 *μ*g/mg extract). Citric acid, kaempferol, and rutin were much more abundant in FVLE at concentrations of 1580.49 ± 79.02, 1208.51 ± 60.43, and 361.66 ± 18.08 *μ*g/mg extract, respectively. Moreover, malic acid was more elevated in FVRE extract (4273.43 ± 213.67 *μ*g/mg extract) compared with FVLE extracts (3240.98 ± 162.05 (*μ*g/mg extract). The variability between phenolic compounds in FVLE and FVRE is attributed to the difference between organs and tissue in a plant to synthesize secondary metabolites such as phenolic compounds. Indeed, the anabolism of phenolic compounds in medicinal plants is depending on tissue specific regulation. This control is mediated by epigenetic factors, in particular DNA methylation, histone modifications, and chromatin compaction [19, 20]. Previous works showed that *F. vulgare* is rich in bioactive compounds including phenolic components [21, 22]. The phenolic composition of *F. vulgare* is not exactly similar to those found in the literature. Indeed, Méabed et al. [21] showed that the predominant phenolic compounds in *F. vulgare* aqueous extracts are ferulic acid, hesperidin, and chlorogenic acid. Moreover, acetone extract of *F. vulgare* showed the presence of palmitic, oleic, and linoleic acid as main components [22]. De Marino et al. [23] have isolated three phenolic glycosides compounds (one benzoisofuranone and two diglucoside stilbene trimers) from *F. vulgare*. This difference may be due to the geographical origins of the plant, environmental stimulus, climatic conditions, and extraction methods, which affect the yield of secondary metabolites [24, 25]. On the contrary, Yaldiz and Camlica [26] have studied the variation in phenolic compounds, fatty acids, and volatile compounds of fruit extracts of different fennel genotypes. They showed a remarkable variability which explains that genotype has an important role in synthesis and secretion of secondary metabolites in medicinal plants.

### 3.2. Antioxidant Activity

Recently, the interest in natural antioxidants has considerably increased worldwide. Plants produce various antioxidants compounds such as phenolic acids, flavonoids, and tannins which have a potent capacity to prevent oxidative stress caused by reactive oxygen species. These compounds have low or no side effects for use in preventive medicine in comparison to the synthetic antioxidant agents. Considering the complexity of the oxidation process, it is necessary to combine the responses of different and complementary tests to evaluate the antioxidant activity of samples. In this study, we evaluated antioxidant capacity of the aqueous extracts of the leaves and rootstocks of *F. vulgare* using the DPPH and ABTS radical scavenging methods.

**Figure 1:**
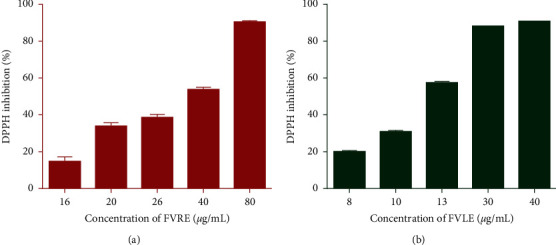
DPPH radical scavenging activity of *F. vulgare* leaf and rootstock aqueous extracts. The values are the mean of three determinations ± standard error.

The DPPH test is often used for the results rapidity as it is used for the screening of molecules with antioxidant activity present in plant extracts. The DPPH is a stable-free radical with a dark purple color; when a DPPH solution is mixed with a substance that can give a hydrogen atom, the color of the reaction mixture changes from purple to yellow with decreasing absorbance at wavelength 517 nm. The DPPH radical scavenging effect of the extracts is shown in Figure 1. The results reveal that the extracts tested have a dose-dependent activity. In fact, at the concentration of 40 *μ*g/mL, the aqueous extracts tested reduce the DPPH radical with an important percentage of 90.94 ± 0.09% and 53.60 ± 1.44% for FVLE and FVRE, respectively. Additionally, the IC_50_ is inversely proportional to the antioxidant capacity of a compound. However, the lowest value of IC_50_ indicates a strong antioxidant capacity of a compound. The IC_50_ values of aqueous extracts of *F. vulgare* are shown in Table 2. The results showed that FVLE (IC_50_ = 12.16 ± 0.02 *μ*g/mL) had better antioxidant activity than that of FVRE (IC_50_ = 34.36 ± 0.09 *μ*g/mL) against the radical DPPH. However, they showed a relatively lower effect than that of Trolox (IC_50_ = 1.47 ± 0.02 *μ*g/mL). This antioxidant power of the aqueous extracts is explained by the presence of phenolic compounds including flavonoids present in the two parts studied and which are known as antioxidant substances with the ability to trap radical species and reactive forms of oxygen.

**Figure 2:**
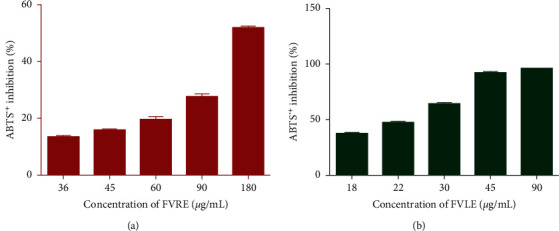
ABTS radical scavenging activity of *F. vulgare* leaf and rootstock aqueous extracts. The values are the mean of three determinations ± standard error.

On the contrary, the antioxidant activity of samples using the ABTS method is deduced from their ability to inhibit the radical cation ABTS^•+^. The ABTS^•+^ radical is in contact with a donor of H^•^ leads, at 734 nm, to ABTS^+^ and to the fading of the solution [27]. The ability of the aqueous extracts to scavenge ABTS^•+^ radical has been illustrated in Figure 2. The percentage inhibition of both extracts increases with increasing concentrations. Indeed, at the concentration of 90 *μ*g/mL, FVLE and FVRE showed significant inhibition of 96.27 ± 0.33% and 27.72 ± 0.80%, respectively. The results show that the FVLE also possesses the most powerful antioxidant activity (IC_50_ = 22.95 ± 0.4 *μ*g/mL) against the ABTS radical (Table 2).

The difference between FVLE and FVRE in the antioxidant effect could be attributed to the variation in the chemical composition. Indeed, several types of bioactive compounds known for their antioxidant activity [28–32] are identified in FVLE with high levels compared to FVRE, including flavonoids (rutin, quercitrin, and kaempferol) and phenolic acids (chlorogenic acid, ferulic acid, and sinapic acid) (Table 1). On the best of our knowledge, no study has been conducted regarding the chemical composition and antioxidant activity of *F. vulgare* rootstock extracts. However, several studies have investigated the antioxidant activity of other parts of this plant, especially the seeds [33–35]. According to a study performed in Italy, the wild *F. vulgare* seeds *n*-Hexane extracts showed an important radical scavenging activity, with an IC_50_ value of 31 *μ*g/mL [36]. Similarly, Anwar et al. [37] showed that the essential oil, ethanol, and methanol extracts of fennel seeds have excellent radical scavenging activity, with IC_50_ values of 32.32, 23.61, and 26.75 *μ*g/mL, respectively. Also, the ethanolic and aqueous seed extracts of *F. vulgare* evaluated by different antioxidant assays presented important antiradical effects [38, 39]. The results of this study suggest the therapeutic interest of this plant and justify the use of its aqueous extract in traditional Moroccan medicine.

### 3.3. Antihyperglycemic Activity

Diabetes mellitus is characterized by high levels of glucose in the blood that can lead to several serious complications [40]. In modern medicine, the treatment of this condition is based on the use of insulin and oral antidiabetic drugs, which have side effects [41]. Medicinal plants and their active components can be a promising source for new safer antidiabetic drugs [42, 43]. Many species of plants are used for this purpose [44]. In the present work, two extracts of *F. vulgare* are evaluated for their *in vitro* antidiabetic activity. The polysaccharides and disaccharides must undergo enzymatic hydrolysis to be converted into monosaccharide absorbable by the enterocytes. The enzymes *α*-amylase and *α*-glucosidase are responsible for the breakdown of carbohydrates in the digestive tract. When starch is ingested, it is hydrolysed in a proportion of 30 to 40% by *α*-amylase present in the saliva. The residual starch is then hydrolysed by pancreatic *α*-amylase into maltose which is converted into glucose by *α*-glucosidases present at the brush border of the intestinal cells. The inhibition of *α*-amylase and *α*-glucosidase activity is therefore an important strategy to limit the postprandial hyperglycemia [45, 46].

**Figure 3:**
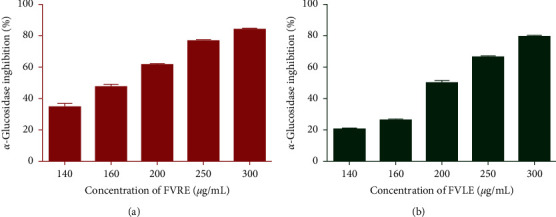
Percentage of *α*-glucosidase inhibition versus different concentrations of *F. vulgare* leaf and rootstock aqueous extracts.

**Figure 4:**
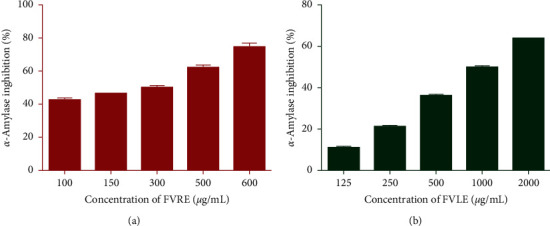
Percentage of *α*-amylase inhibition versus different concentrations of *F. vulgare* leaf and rootstock aqueous extracts.

To determine the *in vitro* antidiabetic activity of aqueous extracts of *F. vulgare* leaves and rootstocks, we tested their inhibitory properties against *α*-amylase and *α*-glucosidase. Indeed, the inhibitory activity of the extracts on *α*-glucosidase has been evaluated at different concentrations and the results are expressed as percentage inhibition (Figure 3). Both extracts showed *α*-glucosidase inhibitory effect depending on the concentration (140 to 300 *μ*g/mL). Furthermore, the inhibitory activity of FVRE was more remarkable (IC_50_ = 165.90 ± 1.2 *μ*g/mL) than that of FVLE (IC_50_ = 203.80 ± 1.3 *μ*g/mL) (Table 3). Moreover, the extracts showed a dose-dependent inhibitory capacity on the *α*-amylase enzyme (Figure 4). Also, FVRE exhibited an inhibitory effect (IC_50_ = 194.30 ± 4.8 *μ*g/mL) greater than that of FVLE (IC_50_ = 1026.50 ± 6.5 *μ*g/mL) and is significantly more potent (*p* < 0.05) than the reference drug acarbose (IC_50_ = 311.20 ± 1.38 *μ*g/mL) (Table 3). The differences between FVLE and FVRE in the enzymes inhibitory action can be attributed to the variations in the percentage of inhibition with respect to the chemical composition of the plant parts as well as the enzymatic sensitivity.

According to the results found, the aqueous extracts of *F. vulgare* demonstrate an inhibitory capacity against two enzymes involved in sugar digestion and, consequently, a capacity to decrease the postprandial hyperglycemia and to prevent type 2 diabetes (T2DM). This confirms that the compounds responsible for the antidiabetic activity of fennel are extractable in water, which explains the use of this herb to treat diabetes in folk medicine. In fact, as mentioned above, several phytochemicals were identified in the extracts studied with different concentrations, especially phenolic acids (quinic acid, chlorogenic acid, and caffeic acid) (Table 1). These compounds have been shown to possess antidiabetic activity attributed to several mechanisms. Effectively, Ooi et al. [47] have reported that 3,4-di-O-caffeoyl quinic acid has an inhibitory effect on *α*-glucosidase. Chlorogenic acid can inhibit the activity of *α*-amylase and *α*-glucosidase and decrease postprandial glucose [48] by reducing glucose transport in a synergistic way [49]. With regard to caffeic acid, it has been able to reduce hyperglycemia and prevent certain complications related to diabetes [50]. An *in vitro* study showed that caffeic acid derivatives could inhibit *α*-amylase, *α*-glucosidase, and angiotensin-converting enzyme related to T2DM [51]. Overall, phenolic compounds have an inhibitory effect on carbohydrate-hydrolyzing enzymes by their protein binding property [52]. According to Jung et al. [53], the antidiabetic power of caffeic acid has been attributed to major mechanisms such as stimulation of insulin production and glucose uptake by adipocytes, reduction of hepatic glucose level, and promotion of antioxidant potential. However, herbal medicine depends on the therapeutic effect of the combination of several compounds that act most often synergistically. It can be deduced that the bioactive compounds of fennel extracts can exert their *in vitro* antidiabetic and antioxidant activities in synergy.

The present data are consistent with two recent studies evaluating the *in vitro* antidiabetic activity of *F. vulgare* on *α*-glucosidase and *α*-amylase [54, 55]. Indeed, Abu-Zaiton et al. [54] found that the aerial parts of this plant had an inhibitory activity of 82.26% and 82.43% on *α*-glucosidase and *α*-amylase, respectively, and the same results were noted for the seeds of three different extracts of *F. vulgare* [55].

On the contrary, several studies have been conducted to evaluate antidiabetic effect of fennel in animal models. Indeed, the aqueous and ethanolic extracts of *F. vulgare* seeds showed correction of hyperglycemia, increased insulin, and improved lipid profile in streptozotocin-induced diabetic rats [56–58]. The essential oils of this plant have also shown a significant hypoglycemic effect in diabetic rats [59, 60]. Interestingly, extracts from other parts of this plant have also been investigated. Indeed, fruits [61], leaves [62], and aerial parts [63] were able to decrease blood glucose levels in diabetic rats and restore other parameters. All these data can explain the use of this herb to treat diabetes in folk medicine. The present study demonstrated the inhibitory effect of aqueous extracts prepared from *F. vulgare* leaves and rootstocks on the activity of *α*-amylase and *α*-glucosidase. To our knowledge, it should be noted that the inhibitory power of *F. vulgare* rootstocks was studied for the first time.

## 4. Conclusion

Both aqueous extracts from the leaf and rootstock of *F. vulgare* are rich in phenolic compounds, particularly phenolic acids. *In vitro* biological investigations showed that *F. vulgare* leaf and rootstock aqueous extracts exhibited important antioxidant and antihyperglycemic effects. Indeed, the rootstock extract (investigated for the first time) revealed important antihyperglycemic effects, which were mediated by the inhibition of enzymes implicated in sugar metabolism (*α*-amylase and *α*-glucosidase). On the contrary, the antioxidant activity of *F. vulgare* leaf and rootstock aqueous extracts can also be useful to improve the management of people with diabetes. These finding suggest that phenolic compounds of *F. vulgare* are responsible for these biological effects. However, further investigations regarding the isolation of these main compounds and evaluation of their antioxidant and antidiabetic activities are required.

## Figures and Tables

**Table 1:** Phenolic composition of *F. vulgare* rootstock and leaf extract.

Phenolic acids	FVLE (*μ*g/mg extract)	FVRE (*μ*g/mg extract)	RT (min)
Quinic acid	14945.61 ± 747.28	29416.42 ± 1470.82	0.51
Malic acid	3240.98 ± 162.05	4273.43 ± 213.67	0.59
Pyrogallol	31.16 ± 1.56	8.32 ± 0.42	0.6
Citric acid	1580.49 ± 79.02	352.06 ± 17.60	0.63
Succinic acid	23.80 ± 1.19	17.35 ± 0.87	0.65
Gallic acid	10.18 ± 0.51	88.73 ± 4.44	0.76
Chlorogenic acid	13810 ± 690.53	5455.98 ± 272.80	0.83
3-4-Hydroxybenzoic acid	1.60 ± 0.08	2.07 ± 0.10	0.95
Pyrocatechol	13.02 ± 0.65	12.17 ± 0.61	1.02
4-Hydroxybenzoic acid	18.72 ± 0.94	25.68 ± 1.28	1.3
3-Hydroxybenzoic acid	44.43 ± 2.22	83.28 ± 4.16	1.34
Catechin	0.58 ± 0.03	0.25 ± 0.01	1.63
Caffeic acid	509203 ± 254.60	2594.24 ± 129.71	1.68
Epicatechin	4.55 ± 0.23	1.10 ± 0.06	1.71
Benzoic acid	4.21 ± 0.21	3.36 ± 0.17	1.8
Epigallocatechin gallate	6.13 ± 0.31	5.24 ± 0.26	2.01
Syringic acid	8.83 ± 0.44	62.09 ± 3.10	2.05
Vanillic acid	8.62 ± 0.43	7.76 ± 0.39	2.12
4-Hydroxycinnamic acid	35.91 ± 1.80	6.76 ± 0.34	2.21
Rutin	361.66 ± 18.08	1.03 ± 0.05	2.63
Sinapic acid	21.00 ± 1.05	4.67 ± 0.23	2.74
3-Hydroxycinnamic acid	0.37 ± 0.02	0.20 ± 0.01	2.98
Ferulic acid	127.36 ± 6.37	65.01 ± 3.25	3.12
Quercetin	7.33 ± 0.37	0.12 ± 0.01	3.48
2-Hydroxycinnamic acid	0.26 ± 0.01	0.15 ± 0.01	3.65
Salicylic acid	5.57 ± 0.28	5.69 ± 0.28	3.68
Naringin	35.90 ± 1.80	15.73 ± 0.79	3.84
Rosmarinic acid	0.44 ± 0.02	0.16 ± 0.01	4.01
Luteolin	10.92 ± 0.55	2.45 ± 0.12	5.01
Resveratrol acid	1.18 ± 0.06	0.27 ± 0.01	5.83
Quercitrin	34.36 ± 1.72	0.65 ± 0.03	5.99
Kaempferol	1208.51 ± 60.43	55.43 ± 2.77	6.01
Hesperetin	Nd	Nd	Nd
Hesperidin	Nd	Nd	Nd
Naringenin	Nd	Nd	nd
4-Hydroxycoumarin	Nd	Nd	Nd
Aesculin	Nd	Nd	Nd
Esculetin	Nd	Nd	Nd
Tannic acid	Nd	Nd	Nd

**Table 2:** IC_50_ values of FV aqueous extracts on DPPH and ABTS scavenging activity.

	DPPH IC_50_ (*μ*g/mL)	ABTS IC_50_ (*μ*g/mL)
FVRE	34.36 ± 0.09^c^	178.45 ± 2.65^c^
FVLE	12.16 ± 0.02^b^	22.95 ± 0.41^b^
Trolox	1.47 ± 0.02^a^	0.68 ± 0.02^a^

The data are the mean of three determinations ± standard error. Values in the same column not sharing a common letter (a to c) differ significantly at *p* < 0.05.

**Table 3:** IC_50_ values of FV aqueous extracts on *α*-amylase and *α*-glucosidase inhibition.

	*α*-Amylase IC_50_ (*μ*g/mL)	*α*-Glucosidase IC_50_ (*μ*g/mL)
FVRE	194.30 ± 4.8^a^	165.90 ± 1.2^b^
FVLE	1026.50 ± 6.5^c^	203.80 ± 1.3^c^
Acarbose	311.20 ± 1.38^b^	18.01 ± 2.00^a^

The values are the mean of three determinations ± standard error. Values in the same column not sharing a common letter (a to c) differ significantly at *p* < 0.05.

## Data Availability

All the data associated with this work are included in the manuscript.

## References

[1] Tang S. Y., Halliwell B. (2010). Medicinal plants and antioxidants: what do we learn from cell culture and *Caenorhabditis elegans* studies?. *Biochemical and Biophysical Research Communications*.

[2] Ghasemi Pirbalouti A. (2009). *Iranian Medicinal and Aromatic Plants*.

[3] Kooti W., Ghasemiboroon M., Asadi-Samani M. (2014). The effect of hydroalcoholic extract of celery leaves on the delivery rate (fertilization and stillbirths), thenumber, weight and sex ratio of rat off spring. *Advances in Environmental Biology*.

[4] Beyrami-Miavagi A., Farokhi F., Asadi-Samani M. (2014). A study of the effect of prostodin and hydroalcoholic extract of *Malva neglecta* on kidney histopathology and renal factors in female rats. *Advances in Environmental Biology*.

[5] Zimmet P., Alberti K. G. M. M., Shaw J. (2001). Global and societal implications of the diabetes epidemic. *Nature*.

[6] El Omari N., Sayah K., Fettach S. (2019). Evaluation of *in vitro* antioxidant and antidiabetic activities of *Aristolochia longa* extracts. *Evidence-Based Complementary and Alternative Medicine*.

[7] Saad F., Mrabti H. N., Sayah K. (2019). Phenolic content, acute toxicity of *Ajuga iva* extracts and assessment of their antioxidant and carbohydrate digestive enzyme inhibitory effects. *South African Journal of Botany*.

[8] Sayah K., Marmouzi I., Naceiri Mrabti H., Cherrah Y., Faouzi M. E. A. (2017). Antioxidant activity and inhibitory potential of *Cistus salviifolius* (L.) and *Cistus monspeliensis* (L.) aerial parts extracts against key enzymes linked to hyperglycemia. *BioMed Research International*.

[9] Bouyahya A., Belmehdi O., El Jemli M. (2019). Chemical variability of *Centaurium erythraea* essential oils at three developmental stages and investigation of their *in vitro* antioxidant, antidiabetic, dermatoprotective and antibacterial activities. *Industrial Crops and Products*.

[10] Lamba S. S., Buch K. Y., Lewis H., Lamba J. (2000). Phytochemicals as potential hypoglycemic agents. *Studies in Natural Products Chemistry*.

[11] Jarald E., Joshi S. B., Jain D. (2008). Diabetes and herbal medicines. *Iranian Journal of Pharmaceutical Research*.

[12] Kashikar V. S., Kotkar T. (2011). Indigenous remedies for diabetes mellitus. *International Journal of Pharmacy and Pharmaceutical Sciences*.

[13] Singh U., Singh S., Kochhar A. (2012). Therapeutic potential of antidiabetic neutraceuticals. *Phytopharmacol*.

[14] Napoli E. M., Curcuruto G., Ruberto G. (2010). Screening the essential oil composition of wild Sicilian fennel. *Biochemical Systematics and Ecology*.

[15] Miraldi E. (1999). Comparison of the essential oils from tenFoeniculum vulgare Miller samples of fruits of different origin. *Flavour and Fragrance Journal*.

[16] Tanira M. O. M., Shah A. H., Mohsin A., Ageel A. M., Qureshi S. (1996). Pharmacological and toxicological investigations onFoeniculum vulgare dried fruit extract in experimental animals. *Phytotherapy Research*.

[17] Marmouzi I., Kharbach M., El Jemli M. (2019). Antidiabetic, dermatoprotective, antioxidant and chemical functionalities in *Zizyphus lotus* leaves and fruits. *Industrial Crops and Products*.

[18] Marmouzi I., Karym E., Saidi N. (2017). *In vitro* and *in vivo* antioxidant and anti-hyperglycemic activities of Moroccan oat cultivars. *Antioxidants*.

[19] Vriet C., Hennig L., Laloi C. (2015). Stress-induced chromatin changes in plants: of memories, metabolites and crop improvement. *Cellular and Molecular Life Sciences*.

[20] Avramova Z. (2015). Transcriptional ’memory’ of a stress: transient chromatin and memory (epigenetic) marks at stress-response genes. *The Plant Journal*.

[21] Méabed E. M. H., El- Sayed N. M., Abou-Sreea A. I. B., Roby M. H. H. (2018). Chemical analysis of aqueous extracts of *Origanum majorana* and *Foeniculum vulgare* and their efficacy on Blastocystis spp . cysts. *Phytomedicine*.

[22] Singh G., Maurya S., De Lampasona M. P., Catalan C. (2006). Chemical constituents, antifungal and antioxidative potential of *Foeniculum vulgare* volatile oil and its acetone extract. *Food Control*.

[23] De Marino S., Gala F., Borbone N. (2007). Phenolic glycosides from *Foeniculum vulgare* fruit and evaluation of antioxidative activity. *Phytochemistry*.

[24] Rodrigues L., Póvoa O., Teixeira G., Figueiredo A. C., Moldão M., Monteiro A. (2013). Trichomes micromorphology and essential oil variation at different developmental stages of cultivated and wild growing *Mentha pulegium* L. populations from Portugal. *Industrial Crops and Products*.

[25] Alipor M., Saharkhiz M. J. (2016). Phytotoxic activity and variation in essential oil content and composition of Rosemary (*Rosmarinus officinalis* L.) during different phenological growth stages. *Biocatalysis and Agricultural Biotechnology*.

[26] Yaldiz G., Camlica M. (2019). Variation in the fruit phytochemical and mineral composition, and phenolic content and antioxidant activity of the fruit extracts of different fennel (*Foeniculum vulgare* L.) genotypes. *Industrial Crops and Products*.

[27] Lien E. J., Ren S., Bui H. H., Wang R. (1999). Quantitative structure-activity relationship analysis of phenolic antioxidants. *Free Radical Biology and Medicine*.

[28] Manach C., Morand C., Texier O. (1995). Quercetin metabolites in plasma of rats fed diets containing rutin or quercetin. *The Journal of Nutrition*.

[29] Chen C. (2016). Sinapic acid and its derivatives as medicine in oxidative stress-induced diseases and aging. *Oxidative Medicine and Cellular Longevity*.

[30] Rice-evans C. A., Miller N. J., Bolwell P. G., Bramley P. M., Pridham J. B. (1995). The relative antioxidant activities of plant-derived polyphenolic flavonoids. *Free Radical Research*.

[31] Boyle S., Dobson V., Duthie S., Hinselwood D., Kyle J., Collins A. (2000). Bioavailability and efficiency of rutin as an antioxidant: a human supplementation study. *European Journal of Clinical Nutrition*.

[32] Torel J., Cillard J., Cillard P. (1986). Antioxidant activity of flavonoids and reactivity with peroxy radical. *Phytochemistry*.

[33] Mohamad R. H., El-Bastawesy A. M., Abdel-Monem M. G. (2011). Antioxidant and anticarcinogenic effects of methanolic extract and volatile oil of fennel seeds (*Foeniculum vulgare*). *Journal of Medicinal Food*.

[34] Roby M. H. H., Sarhan M. A., Selim K. A.-H., Khalel K. I. (2013). Antioxidant and antimicrobial activities of essential oil and extracts of fennel (*Foeniculum vulgare* L.) and chamomile (*Matricaria chamomilla* L.). *Industrial Crops and Products*.

[35] Salami M., Rahimmalek M., Ehtemam M. H. (2016). Inhibitory effect of different fennel (*Foeniculum vulgare*) samples and their phenolic compounds on formation of advanced glycation products and comparison of antimicrobial and antioxidant activities. *Food Chemistry*.

[36] Conforti F., Statti G., Uzunov D., Menichini F. (2006). Comparative chemical composition and antioxidant activities of wild and cultivated *Laurus nobilis* L. leaves and *Foeniculum vulgare* subsp. *piperitum* (Ucria) coutinho seeds. *Biological & Pharmaceutical Bulletin*.

[37] Anwar F., Ali M., Hussain A. I., Shahid M. (2009). Antioxidant and antimicrobial activities of essential oil and extracts of fennel (*Foeniculum vulgare* Mill.) seeds from Pakistan. *Flavour and Fragrance Journal*.

[38] Oktay M., Gülçin İ., Küfrevioğlu Ö. İ. (2003). Determination of *in vitro* antioxidant activity of fennel (*Foeniculum vulgare*) seed extracts. *LWT - Food Science and Technology*.

[39] Chatterjee S., Goswami N., Bhatnagar P. (2012). Estimation of phenolic components and *in vitro* antioxidant activity of fennel (*Foeniculum vulgare*) and Ajwain (*Trachyspermum ammi*) seeds. *Advances in Bioresearch*.

[40] Brownlee M. (1995). The pathological implications of protein glycation,” *Clinical and investigative medicine*. *Medecine Clinique et Experimentale*.

[41] Marles R. J. (1994). Plants as sources of antidiabetic agents. *Economic and Medicinal Plant Research*.

[42] Bnouham M., Mekhfi H., Legssyer A., Ziyyat A. (2002). Ethnopharmacology forum medicinal plants used in the treatment of diabetes in Morocco. *International Journal of Diabetes and Metabolism*.

[43] Kar A., Choudhary B. K., Bandyopadhyay N. G. (2003). Comparative evaluation of hypoglycaemic activity of some Indian medicinal plants in alloxan diabetic rats. *Journal of Ethnopharmacology*.

[44] Rachid A., Rabah D., Farid L., Zohra S. F., Houcine B., Nacéra B. (2012). Ethnopharmacological survey of medicinal plants used in the traditional treatment of diabetes mellitus in the North Western and South Western Algeria. *Journal of Medicinal Plants Research*.

[45] Bhandari M. R., Jong-Anurakkun N., Hong G., Kawabata J. (2008). *α*-Glucosidase and *α*-amylase inhibitory activities of Nepalese medicinal herb Pakhanbhed (Bergenia ciliata, Haw.). *Food Chemistry*.

[46] Malapermal V., Botha I., Krishna S. B. N., Mbatha J. N. (2017). Enhancing antidiabetic and antimicrobial performance of *Ocimum basilicum*, and *Ocimum sanctum* (L.) using silver nanoparticles. *Saudi Journal of Biological Sciences*.

[47] Ooi K. L., Muhammad T. S. T., Tan M. L., Sulaiman S. F. (2011). Cytotoxic, apoptotic and anti-*α*-glucosidase activities of 3,4-di-O-caffeoyl quinic acid, an antioxidant isolated from the polyphenolic-rich extract of *Elephantopus mollis* Kunth. *Journal of Ethnopharmacology*.

[48] Zheng Y., Liu K., Jia G., Li H., Han L., Kimura Y. (2007). Effect of hot-water extract of coffee seeds on postprandial blood glucose concentration in rats. *Chinese Pharmaceutical Journal*.

[49] McCarty M. F. (2005). Nutraceutical resources for diabetes prevention-an update. *Medical Hypotheses*.

[50] Celik S., Erdogan S., Tuzcu M. (2009). Caffeic acid phenethyl ester (CAPE) exhibits significant potential as an antidiabetic and liver-protective agent in streptozotocin-induced diabetic rats. *Pharmacological Research*.

[51] Chiou S.-Y., Sung J.-M., Huang P.-W., Lin S.-D. (2017). Antioxidant, antidiabetic, and antihypertensive properties of *Echinacea purpurea* flower extract and caffeic acid derivatives using *in vitro* models. *Journal of Medicinal Food*.

[52] Shobana S., Sreerama Y. N., Malleshi N. G. (2009). Composition and enzyme inhibitory properties of finger millet (*Eleusine coracana* L.) seed coat phenolics: mode of inhibition of *α*-glucosidase and pancreatic amylase. *Food Chemistry*.

[53] Jung U. J., Lee M.-K., Park Y. B., Jeon S.-M., Choi M.-S. (2006). Antihyperglycemic and antioxidant properties of caffeic acid in db/db mice. *Journal of Pharmacology and Experimental Therapeutics*.

[54] Abu-Zaiton A., Alu’datt M., Wafa M. (2015). Evaluating the effect of *Foeniculum vulgare* extract on enzymes related with blood pressure and diabetes (*in vitro* study). *International Journal of Advances in Chemical Engineering and Biological Sciences*.

[55] Godavari A., Amutha K., Moorthi N. M. (2018). *In-vitro* hypoglycemic effect of *Foeniculum vulgare* mill. Seeds on the carbohydrate hydrolyzing enzymes, alpha-amylase and alpha-glucosidase. *International Journal of Pharmaceutical Sciences and Research*.

[56] Anitha T., Balakumar C., Ilango K. B., Jose C. B., Vetrivel D. (2014). Antidiabetic activity of the aqueous extracts of *Foeniculum vulgare* on streptozotocin-induced diabetic rats. *International Journal of Advances in Pharmacy, Biology and Chemistry*.

[57] Osman N. N., Jambi E. J., Aseri N. H. (2017). Assessment of antidiabetic and antioxidant activities of *Cassia angustifolia* and *Feoniculum vulgare* in diabetic rats. *International Journal of Pharmaceutical Research & Allied Sciences*.

[58] Parsaeyan N. (2016). The effect of *Foeniculum vulgare* (fennel) extract on lipid profile, lipid peroxidation and liver enzymes of diabetic rat. *Iranian Journal of Diabetes and Obesity*.

[59] El-Soud N., El-Laithy N., El-Saeed G. (2011). Antidiabetic activities of *Foeniculum vulgare* Mill. essential oil in streptozotocin-induced diabetic rats. *Macedonian Journal of Medical Sciences*.

[60] Mostafa D. M., Abd El-Alim S. H., Asfour M. H., Al-Okbi S. Y., Mohamed D. A., Awad G. (2015). Transdermal nanoemulsions of *Foeniculum vulgare* Mill. essential oil: preparation, characterization and evaluation of antidiabetic potential. *Journal of Drug Delivery Science and Technology*.

[61] Dongare V., Kulkarni C., Kondawar M., Magdum C., Haldavnekar V., Arvindekar A. (2012). Inhibition of aldose reductase and anti-cataract action of trans-anethole isolated from *Foeniculum vulgare* Mill. fruits. *Food Chemistry*.

[62] El-Ouady F., Lahrach N., Ajebli M., El A. H., Eddouks M. (2019). Antihyperglycemic effect of the aqueous extract of *Foeniculum vulgare* in normal and streptozotocin-induced diabetic rats. *Cardiovascular & Haematological Disorders-Drug Targets (Formerly Current Drug Targets-Cardiovascular & Hematological Disorders)*.

[63] Mhaidat N. M., Abu-zaiton A. S., Alzoubi K. H., Alzoubi W., Alazab R. S. (2015). Antihyperglycemic properties of *Foeniculum vulgare* extract in streptozocin-induced diabetes in rats. *International Journal of Pharmacology*.

